# Patients’ experiences with coercive mental health treatment in Flexible Assertive Community Treatment: a qualitative study

**DOI:** 10.1186/s12888-023-05264-z

**Published:** 2023-10-18

**Authors:** Eva Brekke, Hanne Clausen, Morten Brodahl, Anne S. Landheim

**Affiliations:** 1 Norwegian National Advisory Unit on Concurrent Substance Abuse and Mental Health Disorders, Inland Hospital Trust, Postbox 104, Brumunddal, 2381 Norway; 2 Department of Research and Development, Division of Mental Health Services, Akershus University Hospital, Lørenskog, Norway

**Keywords:** Flexible assertive community treatment (FACT), Coercion, Severe mental Illness, Substance use disorder, Community treatment order, Compulsory medication, Involuntary outpatient treatment, Involuntary inpatient treatment, Patient experiences, Qualitative

## Abstract

**Background:**

Flexible Assertive Community Treatment (FACT) teams have been implemented in Norwegian health and social services over the last years, partly aiming to reduce coercive mental health treatment. We need knowledge about how service users experience coercion within the FACT context. The aim of this paper is to explore service user experiences of coercive mental health treatment in the context of FACT and other treatment contexts they have experienced. Are experiences of coercion different in FACT than in other treatment contexts? If this is the case, which elements of FACT lead to a different experience?

**Method:**

Within a participatory approach, 24 qualitative interviews with service users in five different FACT teams were analyzed with thematic analysis.

**Results:**

Participants described negative experiences with formal and informal coercion. Three patterns of experiences with coercion in FACT were identified: FACT as clearly a change for the better, making the best of FACT, and finding that coercion is just as bad in FACT as it was before. Safety, improved quality of treatment, and increased participation were described as mechanisms that can prevent coercion.

**Conclusion:**

Results from this study support the argument that coercion is at odds with human rights and therefore should be avoided as far as possible. Results suggest that elements of the FACT model may prevent the use of coercion by promoting safety, improved quality of treatment and increased participation.

## Background

Coercive mental health treatment is a controversial issue that presents dilemmas related to human rights, practitioner ethics, and safety. Coercion is at odds with the right to health, human dignity, and freedom. Immediate actions to develop non-coercive practices have been called for, along with research to monitor the progress and facilitate the exchange of good practices [1]. Deinstitutionalization and the development of good-quality, comprehensive, community-based mental health services have been suggested as ways of reforming services to reduce coercive practices [2]. While some countries, such as England and Italy, begun the process of deinstitutionalization more than 40 years ago, progress has been slower in other countries [3]. Limiting the use of coercion and strengthening community based mental health services have been a priority for Norwegian health authorities in the last decades [4]. The implementation in the recent years of Assertive Community Treatment (ACT) and Flexible Assertive Community Treatment (FACT) in Norwegian health care services has partly been aimed at reducing involuntary inpatient treatment [5].

Coercion in Norwegian mental health services is regulated by the Norwegian Mental Health Care Act from 1999 [6]. Coercive treatment is authorized when the person has a severe mental illness and coercion is considered necessary in order to prevent deterioration of the person’s health (the treatment criterion), or because the person may cause serious harm to others or to themselves (the danger criterion). Further, voluntary treatment should always be tried first, unless this is considered to be impossible. Thorough considerations of the negative impact of coercion on the individual must underlie the decision. Since 2017, persons who are considered as having capacity to consent, may only be subjected to coercion based on the danger criterion.

Coercive treatment may be provided in an inpatient mental health facility (involuntary inpatient treatment) or in the community. In the case of community treatment orders (CTOs), the person lives at home and receives involuntary outpatient mental health treatment. Psychiatrists and authorised clinical psychologists make decisions about coercive interventions. A separate medication order is needed if involuntary administration of medication is part of the treatment. Studies have found that between 39 and 44% of Norwegian patients with a CTO had a separate medication order, while more than 98% in the same group were prescribed psychotropic medication [7–9]. Coercive measures are authorized in acute situations and only when necessary. These include mechanical restraint, short-term medication, short-term seclusion and short-term retention. In addition to formal coercion, service users may also experience informal coercive measures, such as the withholding of benefits, persuasion or other forms of pressure [10, 11].

ACT and FACT aim to prevent coercion through integrated, flexible and continuous care using a person-centred and community-based approach [12, 13]. FACT, a European adaptation of ACT, involves a larger target group and flexible changes between intensive and less intensive care provision. The target group for Norwegian FACT teams includes persons with severe psychiatric disorders, with or without co-occurring substance use problems. Additional criteria for inclusion in FACT are the long-term presence of a mental health problem which requires treatment, a severely decreased level of daily functioning, a variation in the need for treatment intensity over time, and a need for co-ordinated services. A focus in client know-how, living conditions, and inclusion in the community are among the cornerstones of the FACT model. The model has been mentioned as an example of good practice in relations to the principles for community-based mental health care suggested by the European Community-based Mental Health Service Providers (EUCOMS) Network, which are human rights, public health, recovery, effectiveness of interventions, community network, and peer support [14].

The first ACT team in Norway was established in 2007, followed by a national investment in establishing ACT and FACT teams since 2009 [15]. The first Norwegian FACT team was established in 2013, and there are now more than 70 FACT teams in Norway distributed in all health regions, in both urban and rural areas.

Patient experiences of coercion in traditional mental health services have been documented in several studies [16, 17]. First-person experiences with coercion are predominantly negative, but a few studies also report perceived positive consequences. Staff behaviour and attitudes, power issues, person-centred service delivery, and environmental factors may affect subjective experiences of coercion [16–19]. Norwegian studies of first-person experiences with involuntary outpatient treatment in traditional services have shown overwhelmingly negative experiences related to coercion, even if some participants positively compared being under a CTO to involuntary inpatient treatment [20, 21]. International research on first-person experiences of CTOs have reported ambivalent and complex experiences such as feelings of coercion and control as well as the CTO as a safety net [22, 23].

Research on ACT has shown that the model was beneficial for client satisfaction and engagement with services among clients who were considered “difficult to engage” [24], and ACT has been shown to reduce involuntary inpatient treatment [25]. Small caseloads, a team approach, frequent encounters and less formal approaches may increase client engagement in ACT compared to regular community mental health care [26]. Qualitative studies of client experiences of coercion in ACT suggest that while coercion is a difficult experience, the model allows for a mutually trusting relationship with staff, useful interventions for everyday struggles and increased participation, leading to a perception of services as less invasive and more helpful [27, 28]. In a user-led interview study of satisfaction with ACT among 70 patients in 12 Norwegian ACT teams, participants under a CTO expressed higher levels of satisfaction with ACT than others, which may be due to negative experiences with services prior to inclusion in ACT. However, the authors warn that a low response rate might have affected the results [29].

Knowledge from traditional services and ACT is highly valuable, but there is a need for more information about the experiences of coercion among service users in FACT. Particularly because FACT, compared with traditional mental health services, includes certain qualities that may affect experiences of coercion. These include flexible changes between high and low intensity, a specific focus on improving living conditions and citizenship, and a wider target group.

Qualitative research has reported that previous experiences of coercion may be a barrier to community participation as well as involvement in treatment and follow-up for service users in FACT [30]. Apart from this, there is little research on how service users in FACT experience coercion. There is little research on service user perspectives on elements of care that may prevent coercion, in FACT or otherwise. We need knowledge of how service users may perceive coercion within the context of FACT. Exploring and systematising the experiences of service users may lead to increased understanding and empathy for a phenomenon that most people will not experience directly [31]. Service users, carers and practitioners experience coercion differently and hold different attitudes towards it [32–34]. Qualitative research into first-person experiences with coercion may complement other sources of knowledge to inform practitioners and decision makers on how to reduce the use of coercion and its negative consequences for the individual.

The aim of this study is to explore service user experiences of coercive mental health treatment in the context of FACT and other treatment contexts they have experienced. Are experiences of coercion different in FACT than in other treatment contexts? If this is the case, which elements of FACT lead to a different experience?

## Methods

### Design

This study has a qualitative, explorative, participatory design, and is part of a larger project, which investigates the implementation of the FACT model in Norway. Other sub-studies of the project have investigated adaptation of the FACT model in the Norwegian service system from the perspectives of staff and collaborating services [35–37], and e-health and Information and Communication Technologies in FACT [38], while the current sub-study explores service user experiences with the FACT model. In a previous publication on the same material, we have investigated experiences with how FACT may support or inhibit citizenship [30]. Additionally, the material comprised information regarding the participants’ experiences with coercion, which forms the material for the current study. The context and methodology have been described in detail in a previous publication [30].

As part of the participatory design, a peer group was consulted at regular meetings with the first and third authors during planning, recruitment, and data collection. Group members are two peer support workers from different FACT teams, one person with service user experience in FACT, and one person who is active in a local peer support house. At a participatory level, the third author, who has lived experience of receiving mental health and substance use services, has participated as a co-researcher in all stages of the study, including planning, data collection and analysis, as described in the following sections.

### Context

This study is based on qualitative interviews with people who received services from five different FACT teams in Norway. Three of the teams were located in rural areas, one team was in a small town, while one team was in a city. The teams followed the FACT model [39] and were interdisciplinary, including a peer support worker, case managers (nurses and social workers), a psychiatrist and a psychologist. Two of the teams had a music therapist. Interviews were conducted between September 2020 and February 2021. COVID-19 restrictions varied between regions and time periods during the data collection, as described below.

### Recruitment

Recruitment was organised by the peer support workers and team leaders in each team. Team members handed out flyers to all service users during a limited time period, with information about the project and contact details of the first and third authors. Some participants contacted us directly, some forwarded their contact details through team members, while others agreed to participate but preferred not to be in touch before the interview. The recruitment strategy aimed at diversity in substance use and mental health problems, experiences of coercion, age, gender, and duration of contact with services. All participants received services from one of the five FACT teams, and had experiences with coercion. Of the 32 interviews conducted in the previous study [30], 24 were selected for analysis in the current study because they contained descriptions of experiences of formal or informal coercion.

### Participants

Participants were six women and 18 men, aged between 26 and 67 years. Sixteen participants had been diagnosed with a psychotic disorder (schizophrenia or schizoaffective disorder), five had a bipolar disorder, and three had other mental health diagnoses. Four participants reported problems with alcohol, ten had problems with other substances, while ten had no substance use problems. The participants had been in contact with FACT for 29 months on average (from four to 78 months). Seventeen participants had experienced involuntary inpatient mental health treatment, and eight of these had been under a CTO. One participant was currently under a CTO. In addition, eight participants described experiences of coercion without a formal decision.

### Data collection

Interviews were held by the first and third authors together. The first author is a psychologist/PhD who has previously worked as a psychologist in two different FACT teams. The second author is a peer researcher who has service user experience from substance use and mental health services, and who has worked as a peer support worker. Interviews were organized as semi-structured conversations based on an interview guide with open-ended questions about participants’ experiences of receiving services from a FACT team. The interview guide was developed based on input from the peer group. Participants were asked about their current status regarding coercion, and whether they had experienced coercion in the past. While the open-ended questions did not specifically address experiences of coercion, the interviews resulted in a nuanced and detailed material about experiences with coercion. In three of the teams, interviews were carried out in person in the participant’s home or on the FACT premises, according to the participant’s preferences. In one team, due to COVID-19 restrictions, interviews were held on a secure digital platform facilitated by a team member. In the last team, interviews were held by telephone because of a severe lockdown preventing team members from arranging digital interviews. In all cases, interviewers offered a short debriefing at the end of the interview. Interviews were tape recorded and transcribed by the first author. One participant did not consent to tape recording, and in this case notes from the interview were included in the analysis. Interviews lasted from 28 to 79 min.

### Ethical considerations

Persons with severe mental illness and substance use problems who are subject to coercive measures, are considered vulnerable groups in research ethics guidelines. It may be discriminating and paternalistic to exclude entire groups from participating in research based on general considerations of vulnerability. It may also lead to a lack of knowledge about the experiences of people from these groups [33, 40]. Groups that are underrepresented in research should be provided appropriate access to participation in research. Research with vulnerable groups may be justified when the research is responsive to the health needs and priorities of this group [41]. Individual issues may however call for the researchers’ particular attention in order to avoid harmful consequences, to ensure informed consent and confidentiality, and to support beneficial consequences of participating in research [42]. One person was subject to a CTO at the time of the interview, and several participants were unsure about the current legal status of their treatment. This situation may increase the likelihood of being wronged or of incurring additional harm. Vulnerable groups should receive specifically considered protection [41], and several measures were taken to ensure that participants did not feel coerced to participate in the interview. It was underlined that interviews were confidential and that they would not affect participants’ future services in any way. Participants could choose whether to inform FACT team members of their decision to participate or not in the study. Informed written consent was a requirement for participation. All participants were considered fully capable of giving consent at the time of the interview. Written and oral information was given to all participants before the interview started, with the opportunity to withdraw at any point. It was underlined that any decision to withdraw from the study would remain confidential.

Any information that could reveal participants’ identity was modified during analysis in order to protect confidentiality. Digital recordings were conducted using a separate recording device, and sound tracks were uploaded to a secure data storage area directly after each interview, and then deleted from the device. After transcription, all sound files were deleted from the secure data storage area. All transcripts and documents from the analysis were saved on a secure data storage area at all times. The study was approved by the local data protection officer (ID 137850) and carried out in accordance with the Helsinki declaration [41], and with the ethical standards of the Norwegian National Committee for Research Ethics in the Social Sciences and Humanities.

### Analysis

The analysis followed the principles of thematic analysis [43]. Firstly, the first author read the transcripts of the recorded interviews several times to enable familiarisation with the data. Secondly, the data set was read through systematically, giving equal attention to each data item, coding meaningful content that was relevant to the research question. When all data had been coded, codes were sorted into two overarching themes: ‘Experiences with coercion’ and ‘What can prevent coercion’. Themes were identified on a semantic level, based on the surface meaning of participants’ descriptions. Codes were then sorted into the overarching themes in a recursive process, where themes and codes were reviewed and defined throughout. At this point, all co-authors met to discuss the themes along with text extracts to illustrate the themes, in order to include different perspectives in the analysis. Thirdly, all collated extracts for each potential theme were read through, and themes were adjusted based on the criteria of internal homogeneity and external heterogeneity. Following this process, the entire data set was read through, considering the validity of the candidate themes in relation to the interviews, including coding of additional data that had been missed during the first coding process. At this point, all co-authors met again to discuss the validity of the themes. Following this, data extracts were organised into coherent accounts with accompanying quotations, and the essence of each theme was identified. This led to the creation of an analytic narrative, which constitutes the Results section of this paper.

## Results

Participants described negative experiences with formal and informal coercion. Three patterns of experiences with coercion in FACT were identified: FACT as clearly a change for the better, making the best of FACT, and finding that coercion is just as bad in FACT as it was before. Safety, improved quality of treatment, and increased participation were described as mechanisms that can prevent coercion.

### Experiences with Coercion

Several participants described negative experiences with coercion in the past, which also affected their relationship with the FACT team. Experiences with coercion had left participants feeling bad about themselves, negatively affecting their feeling of self-worth. Involuntary admissions were described as brutal. Being locked up with other people you don’t know and people who are checking on what you are doing was described as frightening. Bars on the windows, not being able to lock the door to the bathroom, psychotic symptoms, and symptoms of severe mental illness among other inpatients were aspects of inpatient treatment that had provoked fear. One participant had been forcibly admitted after being a victim of domestic violence, which felt like she was being punished instead of the person who had exercised the violence. One person said:

I’ve had experience of those kinds of admissions, how brutal they can be. It really hurt me. (…) Because an involuntary admission, well, that’s no walk in the park.

Some participants had been subject to coercion for long periods of time before getting in contact with FACT. One participant described that he struggled with managing adult life because other people had controlled him for so many years. Another participant who had been admitted to involuntary inpatient treatment for long periods of time since early adulthood, described feelings of anger, frustration, loneliness, loss of freedom, loss of meaning, and eventually resignation. These experiences were impossible to forget. He said:

I’ve just been like a pawn in a game of chess. It was utterly pointless. Finally I gave up and got very depressed while I was in a psychiatric hospital.

Forced medication was described as a negative experience that should be avoided when possible, as it involved an intervention into a person’s body against their will. The system for monitoring community treatment orders before getting in contact with FACT was described as disrespectful and degrading by some participants. One participant felt like he had to prove that he was sane every month. Others felt that practitioners had made them out to be sick unnecessarily. Some said that it was nearly impossible to protest against coercion, because any resistance was interpreted as a sign of delusion:

It’s nearly impossible to persuade the authorities that you shouldn’t be under coerced treatment. Any resistance proves them right.

While coercion was described as an overwhelmingly negative experience, some participants had experienced positive *consequences* of coercion. Forced medication for limited time periods had led to positive consequences when this resulted on voluntary treatment, when medication was tailored to reduce side effects and improve effect, and when participants experienced improvements as a result of medication.

Now I’ve got the medicine, things have turned around completely. (…) And it’s quite tough to get there, because I can see how much help I’ve needed for many years, and how much time I’ve wasted on not getting that help.

Most participants in this study described a change for the better in FACT when it came to experiences with coercion. Services were experienced as less top-down and more focussed on opportunities and resources. Some participants described that FACT had helped them to get out of psychiatric treatment and move into their own home.

There’s definitely a change, and a change for the better (…) Before it was more top-down: “You need to be visited, you need to be checked, you need this and that kind of treatment”. And now it’s more on my terms, you see.

Some participants described that they tried to make the best out of a situation that they could not control. Several participants were unsure whether the current treatment was voluntary or not. Some expressed that they were not in a position to advocate for their rights, due to a lack of resources, distrust in the system, lack of a social network, a bad reputation, or simply their role as a person with severe mental illness. Some described that they consented to the current treatment in order to avoid forced medication, trying to make the best out of the situation. Some participants described that getting treatment from FACT meant that they avoided involuntary admissions, because other practitioners were reassured. One participant said:

I’ve agreed to what they said. So then I don’t have to take forced medication at least.

A few participants described that coercion was just as bad in FACT as it had been before. Even if practitioners in FACT tried to be democratic, the system disturbed the one-on-one support. Some felt that they were fighting an authoritarian system, particularly related to forced medication. One participant had attempted suicide as a protest against forced medication. After the suicide attempt, the forced medication had continued, leading to feelings of despair and resignation. Side effects such as putting on weight, loss of energy, and sleeping too much, were described as having a negative impact on some participants’ lives. Several participants described that they were not themselves when they used medication. Emotional numbness and changed opinions were negative consequences of medication. One participant described that she had quit taking medication because they made her less critical towards unacceptable life circumstances that she wanted to protest against. On person said:

Medicines like that, they change you. You’re not yourself. You’re kind of like a zombie. No happiness and no sadness. And sadness can be good to have as well.

Experiences with coercion had broken down trust to the treatment system and to other people in general. The potential for coercion in FACT led to feelings of distrust and fear. Practitioners using diagnostic terms in written reports had felt like an ambush for one participant, leading to panic because she feared that coercion was around the next bend. Another participant described that the police had come to her home without warning to bring her to involuntary inpatient treatment at the request of practitioners in a former service. This was described as a defining life experience where her ability to trust other people had been broken. It had taken a long time before she could trust practitioners in FACT because of this former experience. Several participants described reservation when it came to reporting psychotic symptoms due to fear of forced medication. One participant said:

They have the authority to have me admitted to hospital by force. And it’s terrifying. Any trust you have is so easily broken.

### What can prevent Coercion?

Feeling safe had prevented coercion for participants in this study, and several participants described the FACT team as *a safety net*. They felt supported by the team and could contact them in case of a relapse. Some participants had no one else to contact in a critical situation. For others, contact between family members and practitioners in FACT was reassuring.

I feel safe with that kind of network around me, you know. In case something happened.

Availability and fast access to help was described as an element of FACT that could prevent coercion. Participants felt that they were taken seriously when they told practitioners in FACT that they started to lose control. This had made them feel understood and respected, which had led to feelings of increased human dignity. Several participants were not used to asking for help and felt they did not deserve it. Necessary help and support provided by practitioners in FACT in the early stages of a relapse, even before participants realized that they were doing worse, had led to feelings of safety and increased trust in practitioners.

I don’t always realize I’m as sick as I actually am, but then they see it right away and you get in touch with the people you need to contact.

Improved quality of treatment had prevented coercion for participants in this study. Easy access to a psychiatrist in the team had led to more tailored medication, less side effects and better treatment effects. Collaboration between the case manager and the psychiatrist meant that the psychiatrist had more relevant information about participants than before, because the case manager knew them so well. Collaboration between the psychologist, the psychiatrist and the case manager had led to more accurate diagnostic assessment based on different aspects of participants’ lives. This had made it easier to consent to treatment because it was more relevant and helpful.

Practitioners’ knowledge about substance use could prevent coercion because it led to a better understanding of complex problems, interventions to address substance use problems, better communication about substance use, and more tailored medication. Several participants expressed that this was an area of improvement for practitioners in FACT. Some described that substance use was “the elephant in the room”, and wished that practitioners would address it more. One participant said:

I have complex problems of mental health and substance use. And not everyone has the knowledge they really should have. That’s a challenge.

Some participants had experienced that when psychiatrists knew that they had been taking drugs, decisions were often based more on conviction and less on professional assessment. One participant pointed out the paradox of focusing on sobriety from substance use, while at the same time prescribing involuntary medication for psychiatric problems.

So it’s almost a kind of paradox, because for some periods I’ve been on 14 medicines a day. And then people go on at me and say: “You have to be sober”.

Having a peer support worker in the team was described as a qualitative improvement in FACT as compared to other services. Contact with the peer support worker could prevent coercion because they understood what it was like and could suggest helpful interventions. One participant described that the peer support worker had helped him make up a schedule when he had a manic episode, which he had found brilliant and very useful. Several participants described that the peer support worker had made them lower their guard and feel more hopeful about their own situation.

There’s something about her being there, like it means something in itself. (…) And it’s much better to play ball with someone who understands what it feels like to be forcibly admitted.

Increased participation and involvement had prevented coercion for participants in this study. Psychoeducation about diagnosis, symptoms, and treatment had enabled some participants to get more involved in their own treatment. Making a crisis plan together with the case manager felt reassuring to other participants, because they knew who to contact in case of a crisis. A user-led bed at the nearest hospital had been helpful to one participant during a holiday when the team was unavailable.

Access to treatment other than medication was mentioned as an element that could prevent coercion. Several participants wished that practitioners in FACT had more knowledge of, and better access to, treatment options that did not involve medication. Some felt that practitioners in FACT supported their wish for treatment without medication, such as psychological treatment. Others expressed frustration because practitioners seemed to think that medication was the only recipe for solving their problems.

Several participants expressed that symptoms of mental health problems, such as suspiciousness and paranoid thoughts, made it difficult to speak their mind. Because of this, it was important that practitioners in FACT actively supported participation. Consultations with the psychiatrist about medication were mentioned as particularly difficult to some participants, because of former experiences with coercion, a lack of trust in the psychiatrist, and preoccupation that what they said would turn out badly for them when it came to medication:

I’ve got that paranoid schizophrenia diagnosis, which gets in the way. It’s hard to speak your mind about things when you’re suspicious.

Another participant said:

There are times when I can’t always say what I mean properly. Or I can’t say anything at all because my tongue just gets tied or something.

## Discussion

This article presents nuanced experiences with coercive mental health treatment in the context of FACT from a service user perspective.

Many participants described negative experiences with mental health services before their contact with FACT, and most participants described a change for the better in terms of coercion after inclusion in FACT. To a certain degree, experiences of coercion in FACT were compared to experiences of coercion from other contexts. This resembles the findings from a study of experiences with CTOs, where participants compared these experiences with involuntary inpatient treatment [21]. The change from a disempowering, illness-focussed approach in previous services to a resource-focussed and empowering approach in FACT, concurs with findings from other studies where experiences of coercion have been related to de-subjectivation and a reduction of persons to “sick patients” [44].

Several participants described that they tried to make the best out of a situation that they could not control. Some participants expressed uncertainty about the level and nature of coercion. Several had consented to take medication in order to avoid coercive measures and involuntary admissions. This is similar to findings from studies with people who are under CTOs [21]. While uncertainty about whether or not one is under a CTO might possibly indicate that coercion is felt to be less invasive, clarity regarding coercive treatment is an obvious patient right. These findings suggest that information about patient and human rights is particularly important for persons with serious mental illness and complex needs, in addition to measures that enable people to stand up for their rights. Other studies have also highlighted a need for information, particularly regarding medication, for people under CTO [20, 45]. If there is uncertainty regarding coercion, various non-coercive measures may seem like coercion.

Results from the current study suggest that experiences of coercion may impact people’s feelings of trust and self-efficacy even after the coercive measures are formally ended, which has also been shown in other studies [46]. A few participants felt that coercion was just as bad in FACT as it had been before. Forced medication was described as an extremely invasive measure associated with brutality, powerlessness, anger, frustration, resignation and even suicidality. The metaphor of feeling like ‘a pawn in a game of chess’ indicates feelings of insignificance, powerlessness and dehumanization. Experiences of compulsory medication as particularly invasive resonate with studies of coercion in traditional inpatient and outpatient treatment [20, 47, 48].

Results suggest that providing a sense of safety through close, continuous, skilled and respectful care and treatment in collaboration with carers may reduce coercion. Previous studies have reported that involuntary inpatient treatment may be experienced by patients as a necessary safety measure [19, 49]. In the current study, FACT was described as providing safety in a way that *prevented* involuntary admissions. Safety in the form of an easily accessible safety net of professional helpers and carers, skilled interventions, a strength focus and mutual trust, led to early intervention in crises, which again prevented coercion.

Better quality of diagnosis and medication through dialogue with the case manager and access to a psychiatrist had led to fewer side effects, more involvement in medication, and less hierarchical services. For some participants, the responsible psychiatrist was not a part of the FACT team, which seemed to reduce their opportunity to be involved in decisions regarding medication. Former research has shown that psychiatrists and specialised psychologists feel more confident in making decisions regarding CTOs within the context of ACT, due to frequent and long-term contact with the person, and shared responsibility with other ACT practitioners [50]. These findings support the concept of binding cooperation and collocation of different expertise, which is central in the FACT model.

Increased client involvement such as crisis plans, psychoeducation, a strength focus and being taken seriously by team members were described as a change for the better. Crisis planning as a method of client involvement may reduce the risk of coercive interventions [51], and several studies have reported that patients wish to discuss with staff how to plan for future situations which may trigger coercion [47]. Poor communication combined with a power imbalance between patients and practitioners may impede an appropriate emphasis on patients’ rights and preferences during involuntary inpatient treatment [17]. Enhanced patient participation and communication, particularly about compulsory medication, is related to patients’ satisfaction and autonomy, and might include debriefing during or after hospitalisation [52–54]. Some participants in this study described inadequate resources to advocate for their rights, due to mental health problems, language barriers, unstable housing, poverty or substance use. Improving living conditions may be an important measure to enhance participation and reduce coercion for people with serious mental illness and complex needs.

The FACT model is community-based, recovery-oriented and person-centred, but may lose some of its potential if the team adopts the traditional psychiatric attitudes of the surrounding system. Such attitudes may include the dominance of a bio-medical understanding of mental health problems, a hierarchical organisational culture characterised by power asymmetries, and the objectification of persons with mental health problems. This resonates with a study which reported that official and legal changes towards a greater degree of service user involvement and autonomy did not lead to changes in the dominant psychiatric paradigm as perceived by service users, leading the authors to conclude that fundamental changes in practice and attitudes among practitioners are needed [55]. Other studies have also shown that experiences of coercion may depend on the behaviour of practitioners, the values of the system, and the client’s relationship with professionals [49, 56–58]. Awareness of ethical challenges in coercion among practitioners may prevent coercive interventions [59], and considerations of patients’ moral evaluations of coercion may lead to a more nuanced and respectful dialogue about coercion [60].

Results from this study suggest that measures should be taken to avoid maintaining traditional attitudes and practices in the implementation of the FACT model into mental health services. Such measures may include consistent focus on recovery and service user involvement, systematic model focus and quality improvement, as well as applying evidence based methods such as the Illness Management and Recovery (IMR), Individual Placement and Support (IPS), and Integrated Dual Disorder Treatment (IDDT). Structural aspects of the FACT model, particularly binding cooperation and colocation, long-term follow up and flexible intensity, seem to reduce the use of coercion in FACT.

### Strengths and limitations

The methods do not allow for an immediate generalization of the results, nor for a comparison between the different geographical and cultural contexts. However, insights from this study may be relevant to other contexts, and may direct future research. One strength of this study is the variation among participants concerning age, gender, mental health problems and co-occurring substance use, and the fact that they lived in five different geographical contexts. Due to the variation among participants, and their direct experiences with phenomena of relevance to the research question, we consider the number of participants to be adequate in this study. While the contexts are varied, the five teams are not representative for all FACT teams in Norway. The FACT model may be implemented in different ways in different countries, for instance regarding target group, legislation, geography, and aspects of the surrounding service system, which may affect the transferability of the results.

Several participants who were interviewed by telephone expressed that this was preferable to meeting someone in person, because it made it easier to express themselves. This concurred with our impression that the telephone interviews yielded deep reflections on participants’ experiences. However, during the telephone interviews, we did not have access to non-verbal information, which may have affected our understanding of the content. The digital interviews were more similar to meeting in person, but without the informal interactions before and after the interview, which may have affected the atmosphere of the interviews.

One strength of this study is its rich and nuanced descriptions of experiences with coercion, and we believe that the participation of a co-researcher with lived experience contributed to this. Previous studies have reported that interviewers with lived experience can make it easier to establish trust during interviews [61], elicit richer descriptions, hence increasing the quality of the data [62], and access information that would otherwise not have been shared. Participants in the current study appreciated that one of the researchers had service user experience. Being two interviewers with different backgrounds also helped us complement each other’s perspectives during the interviews, and to reflect on the interview situation and content after each interview [30]. However, none of the authors have direct experience of coercive mental health treatment.

The interviews were not directed specifically towards coercion, which may be a challenge to the internal validity of the study. However, the fact that participants mentioned coercion without being asked explicitly about it suggests that this issue was important to them. The cross-sectional analysis does not allow for investigation of process, and future research should consider using narrative approaches.

### Conclusions and implications

Results from this study support the argument that coercion is at odds with human rights and therefore should be avoided as far as possible. Results suggest that elements of the FACT model may prevent the use of coercion by promoting safety, improved quality of treatment and increased participation.

## Data Availability

The datasets that underlie this study are available from the corresponding author upon reasonable request, but are not made publicly available due to considerations of confidentiality.

## List of abbreviations

FACT: Flexible Assertive Community Treatment.
ACT: Assertive Community Treatment.
CTO: Community Treatment Order.

## References

[1] Püras, D. Report of the Special Rapporteur on the right of everyone to the enjoyment of the highest attainable standard of physical and mental health. Geneva, United Nations; 2017.

[2] World Health Organization and the Gulbenkian Global Mental Health Platform. Innovation in deinstitutionalization: a WHO expert survey. Geneva, World Health Organization; 2014.

[3] Martinelli A, Iozzino L, Ruggeri M, Marston L, Killaspy H (2019). Mental health supported accommodation services in England and in Italy: a comparison. Soc Psychiatry Psychiatr Epidemiol.

[4] Norwegian Ministry of Health and Care Services. St prp nr 63 (1997-98). Om opptrappingsplan for psykisk helse 1999–2006. Endringer i statsbudsjettet for 1998 [Proposition no 63 (1997–1998). On the Escalation Plan for Mental Health 1999–2006. Changes in the state budget for 1998]. Oslo, Norwegian Ministry of Health and Care Services; 1998.

[5] Helse- og omsorgsdepartementet (Ministry of Health and Care) (2023). Opptrappingsplan for psykisk helse (2023–2033). Meld St.

[6] Norwegian Ministry of Health and Care Services. Psykisk helsevernloven [The Mental Health Care Act]. Lovdata; 1999.

[7] Rugkåsa J, Nyttingnes O, Simonsen TB, Saltyte-Benth J, Lau B, Riley H (2019). The use of outpatient commitment in Norway: who are the patients and what does it involve?. Int J Law Psychiatry.

[8] Riley H, Sharashova E, Rugkåsa J, Nyttingnes O, Christensen TB, Austegard AA (2019). Out-patient commitment order use in Norway: incidence and prevalence rates, duration and use of mental health services from the Norwegian outpatient commitment study. BJPsych Open.

[9] Drivenes K, Bergan S, Saeter O (2016). Drug therapy in patients subject to outpatient compulsory mental healthcare. Eur J Person Centered Healthc.

[10] Rugkasa J, Canvin K, Sinclair J, Sulman A, Burns T (2014). Trust, deals and authority: community mental health professionals’ experiences of influencing reluctant patients. Community Ment Health J.

[11] Szmukler G, Appelbaum PS (2008). Treatment pressures, leverage, Coercion, and compulsion in mental health care. J Mental Health.

[12] Stein LI, Test MA (1980). Alternative to mental hospital treatment: I. conceptual model, treatment program, and clinical evaluation. Arch Gen Psychiatry.

[13] van Veldhuizen JR (2007). FACT: a Dutch version of ACT. Commun Ment Health J.

[14] Keet R, de Vetten-Mc Mahon M, Shields-Zeeman L, Ruud T, van Weeghel J, Bahler M (2019). Recovery for all in the community; position paper on principles and key elements of community-based mental health care. BMC Psychiatry.

[15] Norwegian Ministry of Health and Care Services. St meld nr 47 (2008-2009). Samhandlingsreformen. Rett behandling – på rett sted – til rett tid [White paper no 47 (2008-2009). The coordination reform. Proper treatment - at the right place and right time]. Oslo, Norwegian Ministry of Health and Care Services; 2009.

[16] Tingleff EB, Bradley SK, Gildberg FA, Munksgaard G, Hounsgaard L (2017). Treat me with respect. A systematic review and thematic analysis of psychiatric patients’ reported perceptions of the situations associated with the process of coercion. J Psychiatr Ment Health Nurs.

[17] Sugiura K, Pertega E, Holmberg C (2020). Experiences of involuntary psychiatric admission decision-making: a systematic review and meta-synthesis of the perspectives of service users, informal carers, and professionals. Int J Law Psychiatry.

[18] Aguilera-Serrano C, Guzman-Parra J, Garcia-Sanchez JA, Moreno-Küstner B, Mayoral-Cleries F (2018). Variables Associated with the subjective experience of coercive measures in Psychiatric inpatients: a systematic review. Can J Psychiatry.

[19] Larsen IB, Terkelsen TB (2014). Coercion in a locked psychiatric ward: perspectives of patients and staff. Nurs Ethics.

[20] Stensrud B, Høyer G, Granerud A, Landheim AS (2015). Life on hold: a qualitative study of patient experiences with outpatient commitment in two Norwegian counties. Issues Ment Health Nurs.

[21] Riley H, Høyer G, Lorem GF (2014). When Coercion moves into your home’ – a qualitative study of patient experiences with outpatient commitment in Norway. Health Soc Care Commun.

[22] Corring D, O’Reilly R, Sommerdyk C (2017). A systematic review of the views and experiences of subjects of community treatment orders. Int J Law Psychiatry.

[23] Newton-Howes G, Banks D (2014). The subjective experience of community treatment orders: patients’ views and clinical correlations. Int J Soc Psychiatry.

[24] Killaspy H, Bebbington P, Blizard R, Johnson S, Nolan F, Pilling S (2006). The REACT study: randomised evaluation of assertive community treatment in north London. BMJ.

[25] Clausen H, Landheim A, Odden S, Saltyte Benth J, Heiervang KS, Stuen HK (2016). Hospitalization of high and low inpatient service users before and after enrollment into Assertive Community Treatment teams: a naturalistic observational study. Int J Ment Health Syst.

[26] Killaspy H, Johnson S, Pierce B, Bebbington P, Pilling S, Nolan F (2009). Successful engagement: a mixed methods study of the approaches of assertive community treatment and community mental health teams in the REACT trial. Soc Psychiatry Psychiatr Epidemiol.

[27] Stuen HK, Rugkasa J, Landheim A, Wynn R (2015). Increased influence and collaboration: a qualitative study of patients’ experiences of community treatment orders within an assertive community treatment setting. BMC Health Serv Res.

[28] Thøgersen MH, Morthorst B, Nordentoft M (2010). Perceptions of Coercion in the community: a qualitative study of patients in a Danish Assertive Community Treatment Team. Psychiatr Q.

[29] Lofthus AM, Westerlund H, Bjorgen D, Lindstrom JC, Lauveng A, Clausen H (2016). Are users satisfied with Assertive Community Treatment in Spite of Personal restrictions?. Community Ment Health J.

[30] Brekke E, Clausen HK, Brodahl M, Lexén A, Keet R, Mulder CL et al. Service user experiences of how flexible Assertive Community Treatment May support or inhibit citizenship: a qualitative study. Front Psychol. 2021;12. 10.3389/fpsyg.2021.727013 PMC8457351 34566813

[31] Natvik E, Moltu C (2016). Just experiences? Ethical contributions of phenomenologically-oriented research. Scandinavian Psychol.

[32] Lydersen GS, Morken M, Aasland O, Pedersen R, Husum TL (2022). Tvang i psykisk helsevern: En sammenligning av holdninger blant pasienter og ansatte [Coercion in mental health services: a comparison of attitudes between patients and employees]. Tidsskrift for Norsk Psykologforening.

[33] Stensrud B. Experiences with outpatient commitment orders from the perspectives of patients, relatives and staff. UiT - The Arctic University of Norway; 2016.

[34] Canvin K, Rugkåsa J, Sinclair J, Burns T (2014). Patient, psychiatrist and family carer experiences of community treatment orders: qualitative study. Soc Psychiatry Psychiatr Epidemiol.

[35] Trane K, Aasbrenn K, Rønningen M, Odden S, Lexén A, Landheim A (2022). Integration of Care in Complex and Fragmented Service systems: experiences of staff in flexible Assertive Community Treatment teams. Int J Integr Care.

[36] Trane K, Aasbrenn K, Rønningen M, Odden S, Lexén A, Landheim A (2021). Flexible assertive community treatment teams can change complex and fragmented service systems: experiences of service providers. Int J Ment Health Syst.

[37] Trane K, Aasbrenn K, Rønningen M, Odden S, Lexén A, Landheim AS. Flexible Assertive Community Treatment in Rural and remote areas: a qualitative study of the challenges and adaptations of the Model. Front Public Health. 2022;10. 10.3389/fpubh.2022.913159 PMC9379540 35983354

[38] Bønes E, Granja C, Solvoll T (2023). Experiences and Expectations of Information and Communication Technologies in Flexible Assertive Community Treatment teams: qualitative study. JMIR Form Res.

[39] van Veldhuizen JR, Bahler M (2013). Flexible Assertive Community Treatment: Vision, model, practice and organization.

[40] Smith LJ (2008). How ethical is ethical research? Recruiting marginalized, vulnerable groups into health services research. J Adv Nurs.

[41] World Medical Association. WMA Declaration of Helsinki - Ethical Principles for Medical Research Involving Human Subjects. World Medical Association, Helsinki; 1964.

[42] Kvale S, Brinkmann S, InterViews (2009). Learning the craft of qualitative research interviewing.

[43] Braun V, Clarke V (2006). Using thematic analysis in psychology. Qualitative Res Psychol.

[44] Verbeke E, Vanheule S, Cauwe J, Truijens F, Froyen B (2019). Coercion and power in psychiatry: a qualitative study with ex-patients. Soc Sci Med.

[45] Canvin K, Bartlett A, Pinfold V (2002). A ‘bittersweet pill to swallow’: learning from mental health service users’ responses to compulsory community care in England. Health Soc Care Commun.

[46] Wergeland NC, Fause Ã, Weber AK, Fause ABO, Riley H (2022). Increased autonomy with capacity-based mental health legislation in Norway: a qualitative study of patient experiences of having come off a community treatment order. BMC Health Serv Res.

[47] Krieger E, Moritz S, Weil R, Nagel M (2018). Patients’ attitudes towards and acceptance of Coercion in psychiatry. Psychiatry Res.

[48] Nyttingnes O, Ruud T, Rugkåsa J (2016). It’s unbelievably humiliating’-Patients’ expressions of negative effects of Coercion in mental health care. Int J Law Psychiatry.

[49] Valenti E, Giacco D, Katasakou C, Priebe S (2014). Which values are important for patients during involuntary treatment? A qualitative study with psychiatric inpatients. J Med Ethics.

[50] Stuen HK, Landheim A, Rugkåsa J, Wynn R (2018). How clinicians make decisions about CTOs in ACT: a qualitative study. Int J Mental Health Syst.

[51] Molyneaux E, Turner A, Candy B, Landau S, Johnson S, Lloyd-Evans B (2019). Crisis-planning interventions for people with psychotic Illness or bipolar disorder: systematic review and meta-analyses. BJPsych Open.

[52] Katsakou C, Bowers L, Amos T, Morriss R, Rose D, Wykes T (2010). Coercion and treatment satisfaction among involuntary patients. Psychiatric Serv.

[53] Lorem GF, Frafjord JS, Steffensen M, Wang CEA (2014). Medication and participation: a qualitative study of patient experiences with antipsychotic Drugs. Nurs Ethics.

[54] Staniszewska S, Mockford C, Chadburn G, Fenton SJ, Bhui K, Larkin M (2019). Experiences of in-patient mental health services: systematic review. Br J Psychiatry.

[55] Ridley J, Hunter S. Subjective experiences of compulsory treatment from a qualitative study of early implementation of the Mental Health (Care & Treatment) (Scotland) Act 2003. Health & Social Care in the Community. 2013;21(5):509–18. 10.1111/hsc.12041 23551766

[56] Hughes R, Hayward M, Finlay WML (2009). Patients’ perceptions of the impact of involuntary inpatient care on self, relationships and recovery. J Mental Health.

[57] Gault I, Gallagher A, Chambers M (2013). Perspectives on medicine adherence in service users and carers with experience of legally sanctioned detention and medication: a qualitative study. Patient Prefer Adherence.

[58] Stanhope V, Marcus S, Solomon P (2009). The Impact of Coercion on Services from the Perspective of Mental Health Care Consumers with Co-occurring disorders. Psychiatric Serv.

[59] Hem MH, Gjerberg E, Husum TL, Pedersen R (2018). Ethical challenges when using Coercion in mental healthcare: a systematic literature review. Nurs Ethics.

[60] Lorem GF, Hem MH, Molewijk B (2015). Good Coercion: patients’ moral evaluation of Coercion in mental health care. Int J Ment Health Nurs.

[61] Veseth M, Moltu C, Svendsen TS, Nesvåg S, Slyngstad TE, Skaalevik AW (2019). A stabilizing and destabilizing Social World: Close relationships and recovery processes in SUD. J Psychosoc Rehabil Ment Health.

[62] Barber R, Beresford P, Boote J, Cooper C, Faulkner A (2011). Evaluating the impact of service user involvement on research: a prospective case study. Int J Consumer Stud.

